# *Pediococcus acidilactici* P25 Protected *Caenorhabditis elegans* against Enterotoxigenic *Escherichia coli* K88 Infection and Transcriptomic Analysis of Its Potential Mechanisms

**DOI:** 10.1155/2020/7340312

**Published:** 2020-03-30

**Authors:** Keqin Tan, Dun Deng, Xianyong Ma, Yiyan Cui, Zhimei Tian

**Affiliations:** Institute of Animal Science, Guangdong Academy of Agricultural Sciences, State Key Laboratory of Livestock and Poultry Breeding, The Key Laboratory of Animal Nutrition and Feed Science (South China) of Ministry of Agriculture, Guangdong Key Laboratory of Animal Breeding and Nutrition, Guangdong Engineering Technology Research Center of Animal Meat Quality and Safety Control and Evaluation, Guangzhou, China 510640

## Abstract

Enterotoxigenic *Escherichia coli* (ETEC) K88 is a zoonotic pathogen. Previous studies have shown that lactic acid bacteria (LAB) have great potential in promoting health and resisting pathogenic infections; however, relatively little research has been done on the *Pediococcus* genus of LAB. This study is aimed at exploring the mechanisms imparted by *Pediococcus acidilactici* P25 against ETEC K88 in *Caenorhabditis elegans*. The probiotic performance of P25 was investigated in vitro. Colonization of K88 in the intestinal tract of *C*. *elegans* and abundance of enterotoxin genes were measured. In addition, the transcriptome of *C*. *elegans* infected by K88 was analyzed. The result showed that P25 possessed the ability to produce acid, as well as high tolerances to acidic and high bile salt concentrations. Coculture revealed that the growth of ETEC K88 was significantly inhibited by the presence of P25. The median survival of *C*. *elegans* fed P25 was 2 days longer than the group infected with K88 alone (*P* < 0.01). At the same time, the number of colonizing K88 and the abundances of *estB* and *elt* were reduced by up to 71.70% and 2.17 times, respectively, by P25. Transcriptome data indicated that P25 affected expression of genes relative to innate immune response and upregulated the abundance of genes in multiple pathways of *C*. *elegans*, including peroxisome, longevity, and mitogen-activated protein kinase (MAPK) pathways. These results demonstrated that in the presence of P25, K88 colonization and their expression of enterotoxin genes were reduced. This was accomplished through the alteration of environmental parameters (pH and bile salt) as well as through the promotion of the innate immune response processes, increased longevity, and increased antipathogenic bacteria-related pathways. This work highlights the potential application of *P*. *acidilactici* P25 as a probiotic resistant to ETEC K88.

## 1. Introduction

ETEC K88 is a pathogenic variant of *E*. *coli* defined by production of heat-labile (LT) and heat-stable (ST) enterotoxins that can cause diarrheal diseases mainly in newborns and piglets [1, 2]. When K88 infect a host, they colonize the cell surface of the gastrointestinal tract by attaching via pilus (fimbria) and nonpilus adhesin to specific receptors on the microvilli of the small intestine, and LT and ST enterotoxins are produced. Enterotoxins destroy the intestinal cell electrolyte homeostasis, resulting in the loss of fluid and ultimately to secretory diarrhea [3, 4]. The morbidity and mortality of ETEC-caused diarrhea are extremely high. In 2016, nearly 4.2% of all children under 5 years old were infected by ETEC worldwide [5–7]. The death of postweaning diarrhea (PWD) caused by ETEC K88 is also acute. In China, the USA, and the Netherlands, the annual averages of mortality of PWD cases were up to 15%, 15.5%, and 11.5%, respectively. The direct economic loss reached more than 145 million US dollars [8]. Antibiotics are the most common treatment for bacterial infectious diseases, but problems such as drug resistance and bacterial resistance caused by their overuse are reducing their efficacy and are therefore receiving increased attention. Some antibacterial compounds and natural bacteriostats that are candidates for application as antibiotics, such as diaryl (heteroaryl) ketone scaffold (pestalone, pyrrolomycin J), 2-salicyloylbenzofurans (2-(4-methoxyphenyl) benzofuran, 2-(4-methoxyphenyl) benzofuran-6-ol), and plant extracts (essential oils, plumbagin), are gradually being put into use [9–11]; however, their relatively low antibacterial effects, uncertain safety, and expensive costs limit their application. Therefore, the development of more effective replacements for antibiotics is imperative to the treatment and prevention of diarrheal diseases.

Lactic acid bacteria (LAB) consist of four primary genera: *Lactobacillus*, *Leuconostoc*, *Pediococcus*, and *Streptococcus* [12], and most are safe, nontoxic, and not pathogenic to humans [13]. Numerous studies had found that LAB can colonize the inner wall of the digestive tract, improve the digestion of food and immunity, resist erosion by harmful bacteria, maintain the balance of intestinal microecology, and improve oxidative stress resistance. Therefore, they are considered an ideal substitute that promotes health and resistance to infection by pathogen [14–18]. The antimicrobial mechanisms of LAB can be summarized as follows: (1) competing for receptor sites and nutrients in the intestinal tract of the host using surface-specific structures similar to those of the pathogen [19]; (2) generating an unsuitable environment for the growth of pathogenic bacteria, such as highly acidic, hypoxic, and/or hypertonic environments [20, 21]; (3) producing bacteriocin, pediocin, or bacteriocin-like antibacterial compounds to inhibit colonization by pathogenic bacteria; and (4) enhancing anti-inflammatory responses and immune system responses of animals to pathogen [22].

*Caenorhabditis elegans* has relatively high longevity, rapid reproduction rate, and obvious morphological differences between developmental stages, so it is widely used in life science research in areas such as aging mechanisms, animal stress, and natural drug screening [23–25]. Since live bacteria can influence nematode physiology through their metabolites, they can play an important role in controlling lifespan, growth, and reproduction [26–29]. Due to the high level of homology and extensive similarities in metabolism between *C*. *elegans* and human genes [30–32], *C*. *elegans* has been developed as a valuable model host system for an ever-expanding array of microorganisms. Over the last few decades, the use of this nematode model has contributed greatly to the progress of research into the probiotic characteristics of LAB [33, 34]. Due to the highly conserved innate immune- and longevity-regulating systems of this nematode, apparent in pathways such as its mitogen-activated protein kinase (MAPK) pathway and insulin/insulin-like growth factor-1 signaling (IIS) pathway [35–37], it is the perfect model for the study on LAB and intestinal pathogenic microorganisms [38, 39].

In the present work, we explored the resistance mechanisms of *Pediococcus acidilactici* P25 against ETEC K88 both in vivo and in vitro; to do this, we monitored the abundance of enterotoxin genes in ETEC K88 and changes in gene expression of the *C*. *elegans* that were protected by P25.

## 2. Materials and Methods

### 2.1. *C*. *elegans*, Bacterial Strains, and Growth Conditions

In this study, the SS104 strain of *C*. *elegans* harboring the temperature-sensitive allele *glp-4* (*bn2*) l and *E*. *coli* OP50 was provided by the *Caenorhabditis* Genetics Center (CGC), University of Minnesota, Minneapolis, MN, USA. ETEC K88 (CVCC 225) strain of serotype O149:K91,K88ac was purchased from General Microbiological Culture Collection Center, Beijing, China. ETEC K88 (*estB^−^*, *elt^−^*) is the mutant strain of ETEC K88 (CVCC 225) from which the coding genes *estB* and *elt* had been removed via Crispr-Cas9. *P*. *acidilactici* P25 was isolated from fermented tapioca and saved in our lab. ETEC K88 and OP50 were cultivated in Luria-Bertani (LB) (1% tryptone, 0.5% yeast, and 0.5% NaCl; pH 7.0). *P*. *acidilactici* P25 was cultivated in MRS medium (HKM, Guangzhou, China).

SS104, which is able to produce progeny at 15°C but not at 25°C, was fed with *E*. *coli* OP50. SS104 were grown in nematode growth medium (NGM) agar (50 mM NaCl, 1.7% agar, 0.25% peptone, 1 mM CaCl_2_, 5 *μ*g/mL of cholesterol, 1 mM MgSO_4_, and 25 mM KPO_4_ in dH_2_O) plates. The specification of cultures and synchronization of worms were performed according to previously established guidelines [40].

### 2.2. Evaluation of the Probiotic Performance of *P*. *acidilactici* P25 In Vitro

The ability of *P*. *acidilactici* P25 to inhibit ETEC K88 in vitro was measured using the double-layer agar plate diffusion method. About 10 mL of sterilized water medium containing 2% agar was added to the sterilized 9 cm culture dish; after solidifying, sterilized Oxford cups were evenly placed on the agar plate. Then, 100 *μ*L of freshly cultured K88 suspension (10^6^ CFU/mL) was mixed with 20 mL of 0.8% LB agar medium at 50~60°C which was poured onto the dish (outside Oxford cups); after waiting for 1 h, the Oxford cups were removed. 100 *μ*L of P25 fermentation broth supernatant (FB), acid-free P25 FB (adjusted to pH = 5.0 with 2 M NaOH), DL-lactic acid (DL-LA) (pH = 5.0), MRS medium (pH = 5.0), 50 *μ*g/mL ampicillin, H_2_O_2_-free P25 FB (removal of H_2_O_2_ using catalase at pH = 7.2), and 2% H_2_O_2_ was added to each well in the medium. The prepared medium was immediately placed in 4°C and allowed to diffuse for 3~5 hours before the residual liquid in the wells was discarded; the prepared infused agar was incubated at 37°C for 12 h. The diameter of the zones of inhibition was measured with vernier calipers, at an accuracy of 0.01 mm, with three replicates per treatment.

Evaluation of the acid production capacity of P25 was conducted as follows. A P25 suspension (OD_600_ = 0.5) was inoculated into acid-producing medium (1% tryptone, 0.5% yeast extract, 2% anhydrous glucose, 0.001% MgSO_4_·7H_2_O, 0.001% MnSO_4_·H_2_O, 0.001% FeSO_4_·7H_2_O, 0.5% NaCl, and 1 mL Tween-80, pH = 7.5), cultured at 37°C for 12~24 hours, and shaken to determine the pH of FB.

Acid and bile salt tolerance was determined according to methods used in previous research [41, 42] with modifications. The P25 suspension with OD_600_ = 0.5 was inoculated in MRS at pH = 2.5 and at a proportion of 100 : 1 and cultured at 37°C for 2 h, then centrifuged at 10,000 rpm for 2 min; the supernatant was removed, and the cells were resuspended in MRS medium at pH = 6.4. Gradient dilutions were performed in 0.9% normal saline (NS) and plated on MRS agar plates in triplicate followed by incubation at 37°C for 20 h preceding the viability count. The P25 suspension with OD_600_ = 0.5 in MRS medium at pH = 6.4 was used as a control. Similarly, the bile salt tolerance of P25 in 0.3% and 0.6% pig bile salt was measured and calculated as acid tolerance.

### 2.3. Lifespan Assay of *C*. *elegans*

The death model of K88 was established following the methods of previous studies [43] with slight modifications. Overnight cultures of ETEC K88 were centrifuged at 10,000 × g for 2 min, and the supernatant was removed. Cells were resuspended with S-medium (0.585% NaCl, 0.1% K_2_HPO_4_, 0.6% KH_2_PO_4_, 0.1% 5 mg/mL cholesterol (dissolved in ethanol), 1% 1 M C_6_H_5_K_3_O_7_·H_2_O pH = 6, 1% trace metal solution (0.186% EDTA, 0.069% FeSO_4_·7H_2_O, 0.02% MnCl_2_·4H_2_O, 0.029% ZnSO_4_·7H_2_O, and 0.0025% CuSO_4_·5H_2_O), 0.3% 1 M CaCl_2_, and 0.3% 1 M MgSO_4_) three times. K88 cell suspensions (5 × 10^8^~2 × 10^9^ CFU/mL) were added into 24-well (2 mL/well) plates in triplicate. The synchronized nematode eggs were transferred to NGM agar with OP50 at 25°C for 48~60 hours until L4 stage, then transferred to the prepared 24-well plates (18~25 worms/well) containing K88 cells using a foil wire picker, and cultured at 25°C. To monitor *C*. *elegans* lifespan, the number of living nematodes was recorded every day (the transfer of nematodes to the K88 suspension was considered on day 0) until all nematodes had died. Worms fed OP50 or K88 (*estB^−^*, *elt^−^*) (2 × 10^9^ CFU/mL) served as negative controls. A worm was considered dead when it repeatedly failed to respond to touch.

To observe the protective effect of *P*. *acidilactici* P25 towards *C*. *elegans* infected with K88, the worms incubated with P25 (OD_600_ = 0.3, about 10^8^ CFU/mL) at 25°C for 20 h were collected and washed six times in 1 mL S-medium. Surface bacteria were removed from the nematodes via centrifugation and suspension; then, they were transferred to the K88 treatment (PR). The worms treated with OP50 served as negative controls (CK) while attacked group (AT) worms were treated with OP50 for 20 h; then, as much bacteria as possible were removed from the body surface and gut by submerging in 1 mL M9 medium (0.3% KH_2_PO_4_, 0.6% Na_2_HPO_4_, 0.5% NaCl, and 0.1% 1 M MgSO_4_) containing 20 *μ*g/mL levamisole suspension for one minute, six times, and transferred to the K88 treatment. The number of living worms was recorded daily for monitoring lifespan as before.

### 2.4. Bacterial Colonization Assay of the *C*. *elegans* Intestine

The numbers of K88 and P25 cells in the nematodes' intestine were determined according to a modified version of a method described previously [44–46]. Synchronized nematodes were cultured using a 6-well plate, and 10 worms were collected on different days into 1.5 mL sterile Eppendorf tubes. The worms were washed six times with M9 medium containing 25 *μ*g/mL gentamicin before the supernatant was removed and appropriate M9 medium was added to the tubes and vortexed for several times. A moderate amount of liquid was taken from the tubes and dropped into EMB (HKM, Guangzhou, China) (for K88) and MRS (for P25) media. After culturing at 37°C for 20 h, the bacteria on the surface of the nematodes were counted (*N*). Approximately 400 mg SiC particles (1 mm, BioSpec Products, Bartlesville, OK) were added to tubes and vortexed at maximum speed for 15 minutes to disrupt the worms. The suspension was gradient diluted and plated onto EMB or MRS medium to determine the bacteria of the worm's gut (*M*). The number of bacteria colonizing the enteric canal of the worms was calculated with the following formula: colonized bacteria = *M* − *N*.

### 2.5. Mixed Culture of ETEC K88 and *P*. *acidilactici* P25 In Vitro

The mixed-culture method described in previous research was used [21]. Overnight cultures of K88 and P25 were adjusted to 10^8^ CFU/mL and centrifuged at 10,000 × g for 2 min. The supernatant was removed, and 0.9% NS was added to resuspend bacterial cells. Bacterial cells were diluted to 10^6^ CFU/mL by MRS : LB = 1 : 1 (*v*/*v*) medium; then, K88 was coincubated with an equal volume of P25 at 37°C; then, bacterial cells were collected at different times. Separately cultured P25 and K88 served as controls for the mixed cultures. Gradient dilutions were performed in 0.9% NS, plated on EMB or MRS agar plates in triplicate, and incubated at 37°C for 20 h for viability counts.

### 2.6. RNA Extraction and cDNA Synthesis

The RNA extraction method for K88 in the mixed-culture experiments was as follows: bacterial solution was collected into RNase-free centrifuge tubes and centrifuged at 10,000 × g for 2 min, and the medium was removed. Bacterial cells were rinsed three times with 1 mL ice-cold 0.9% NS, lysed with 1 mL TRIpure Reagent (Aidlab Biotechnologies, Co. Ltd., Beijing, China), and vortexed at 5,000 rpm for 5 min at room temperature. Cell lysates stood at 4°C for 5 min, and 250 *μ*L of chloroform was added, after shaking for 1 min, and stood at 4°C for 3 min. Cell lysates were centrifuged at 12,000 rpm at 4°C for 15 min. The aqueous phase was collected, gently mixed with 600 *μ*L isopropanol, and allowed to stand at 4°C for 30 min, then centrifuged at 12,000 rpm at 4°C for 15 min. The supernatant was carefully removed, and 1 mL 75% ethanol was added (in DEPC-treated water) to the gelatinous RNA pellets. Tubes were gently shaken twice to wash the RNA pellets and centrifuged at 12,000 rpm at 4°C for 5 min before the supernatant was discarded. Tubes were allowed to air-dry in the fuming cupboard for 5 min to remove the residual liquid and dissolve the RNA pellets in RNase-free water. RNA integrity was detected by agarose gel electrophoresis. The conditions are as follows: 100 V, 400 mA, and 30 min. The concentration of total RNA was determined with a NanoDrop ND-1000 spectrophotometer (NanoDrop Technologies, Wilmington, USA).

The method of extracting K88 RNA from nematodes in vivo was the same as previously described but with some modifications. The previous method was used to clean the bacteria from the surface of nematodes and break up the worms. 500 *μ*L 0.9% NS was added to the schizolytic worms and centrifuged at 1,000 rpm for 30 sec to isolate the nematode debris from the bacterial solution. The upper layer of bacterial liquid was transferred to new RNase-free tubes for the subsequent RNA extraction steps.

The synthesis of cDNA was performed according to PrimeScript™ RT Reagent Kit with gDNA Eraser protocol (Takara Bio Inc., Dalian, China). The extracted RNA was mixed with 2 *μ*L 5× gDNA Eraser Buffer and 1 *μ*L gDNA Eraser, and RNase-free dH_2_O was added to the total reaction volume of 10 *μ*L. The reaction mixture was then incubated at 42°C for 2 min; then, 1 *μ*L PrimeScript RT Enzyme Mix I, 1 *μ*L RT Primer Mix, 4 *μ*L 5×PrimeScript Buffer 2, and 4 *μ*L RNase-free dH_2_O were added to the reaction tubes for the next incubation at 37°C for 15 min, then 85°C for 5 sec.

### 2.7. Quantitative RT-PCR (qRT-PCR) Assay of ETEC K88 Virulence Gene Abundance

qRT-PCR assays were performed using the C1000 Touch thermal cycler and iTaq Universal SYBR Green Supermix (Bio-Rad, Shanghai, China). 2 *μ*L of each cDNA at 100 ng was included in 10 *μ*L 2× iTaq Universal SYBR Green Supermix, 0.8 *μ*L of each primer at 100 nM, and 6.4 *μ*L ddH_2_O. The qRT-PCR programs were included at 95°C for 30 sec and 39 cycles of 95°C for 15 sec, 56°C for 30 sec, and 72°C for 30 sec. The melting curve was from 60~90°C, increasing by 0.5°C/5 sec. The oligonucleotide primer (Table 1) design for the *estB*, *elt*, and *gapA* (housekeeping gene) genes referred to previously was used [47].

qRT-PCR data were analyzed using the 2^-*ΔΔ*Ct^ method to determine the relative abundances (fold changes) of the target genes [48]. Ct values were determined with the Bio-Rad CFX Maestro software (version 1.0) based on a threshold that was defined by the noninformative fluorescent data. *Δ*Ct represents the value after normalizing each target gene with the Ct value of the housekeeping gene. *ΔΔ*Ct represents the *Δ*Ct value of the sample normalized by the normalized *Δ*Ct value. 2^-*ΔΔ*Ct^ was used to represent abundances of the enterotoxin genes related to the housekeeping gene.

### 2.8. Transcriptomic Analysis of *C*. *elegans*

#### 2.8.1. Library Construction, Sequencing, and Illumina Read Processing

Live nematodes were collected from each group on the 12^th^ day of the lifespan assay, and the total RNA was extracted using the TRIpure Reagent according to the manufacturer's instructions as previously described. The integrity of the RNA samples was tested by agarose gel electrophoresis, RNA purity was tested by NanoDrop, Qubit 2.0 was used to assess quantitative accuracy, and Agilent 2100 was used to measure RNA integrity. Eukaryotic mRNA was enriched using oligo (dT) beads, followed by the fragmentation buffer to break the mRNA into short fragments. Using mRNA as a template, a single-strand of cDNA was synthesized using random hexamers; then double-stranded cDNA was synthesized via adding buffer, dNTPs, DNA polymerase I, and RNase H. Purification of double-stranded cDNA was done using AMPure XP beads. The purified double-stranded cDNA was end-repaired, it was A-tailed and ligated to the sequencing linker, and AMPure XP beads were used for fragment size selection. Finally, PCR amplification was performed and the PCR product was purified using AMPure XP beads to obtain the final libraries. The constructed library was initially quantified via Qubit 2.0 and diluted to 1 ng/*μ*L, the insert size was determined using Agilent 2100, and the effective concentrations (>2 nM) of libraries were determined using qRT-PCR to ensure quality of libraries. The different libraries were pooled in the flow cell according to the effective concentrations and the target data volume. After cBot clustering, the Illumina high-throughput sequencing platform (HiSeq/MiSeq) was used for paired-end (PE) sequencing [49]. The obtained raw reads (double-ended sequences) were quality controlled using the Trimmomatic software and evaluated using FastQC software [50]. The adapters, low-quality reads (Q10 > 50%), or too many *N* (undetermined base information > 10%) reads in the original sequencing data were filtered out to obtain clean reads.

#### 2.8.2. Bioinformatics Analysis

The Bowtie 2 software was used to compare the sequencing data to the ribosomal database to remove reads from the alignment. Subsequently, unmapped reads were compared to the nematode reference genome using the transcriptome data comparison software Hisat2, and the alignment of reads with each reference sequence was counted. Saturation and gene coverage of each sample were analyzed to ensure accurate sequencing and gene abundance in genomic analyses. Gene abundances were calculated and normalized to RPKM (reads per kb per million reads) [51]. Hierarchical clustering analysis was performed based on the gene expression represented by the normalized RPKM value; the relationship between samples and genes were hierarchically clustered; and using the R software package (version 3.6.1), a heat map to present clustering results was generated. Principal component analysis (PCA) was performed for comparison between samples while ensuring that as much of the information contained in the original data set was retained as possible. Analysis of differentially expressed genes (DEGs) was conducted using edgeR. Genes with a *P*a value ≤ 0.05 and |Log2FC)| ≥ 1 are considered candidate DEGs; the *P* values were corrected with the multiple hypothesis test using the BH method. Annotation and enrichment analyses were performed based on the results for DEGs which included gene ontology (GO) annotation and enrichment using clusterProfiler (http://www.geneontology.org) and pathway annotation and enrichment in the Kyoto Encyclopedia of Genes and Genomes (KEGG) database (https://www.genome.jp/kegg).

### 2.9. Quantitative RT-PCR Analysis of the Candidate Genes

Total RNA was extracted from *C*. *elegans* from each treatment, using the qRT-qPCR reaction system and program as described previously in these methods. The oligonucleotide primers of eight pairs of candidate genes (*kin-1*, *hsp-70*, *sek-6*, *gpa-12*, *Y105C5A*.*24*, *atf-5*, *ver-3*, and *ras-2*) and reference genes (*β-actin*) are shown in Table 2.

### 2.10. Statistical Analysis

Analyses of acid production and acid and bile salt tolerance experiments of P25 used one-way ANOVA and Least Significant Difference (LSD) method for multiple comparisons in the SPSS software (version 24.0). Colonization, mixed culture, and relative expression of genes were analyzed using Student's *t*-test in the GraphPad Prism software (version 8.0.2), which used the joint hypothesis test to test homogeneity of variance. The statistical analysis of *C*. *elegans* survival was performed using the log-rank test. All data are presented as “mean ± SD.”

## 3. Results

### 3.1. *P*. *acidilactici* P25 Possessed Excellent Probiotic Properties In Vitro

**Figure 1:**
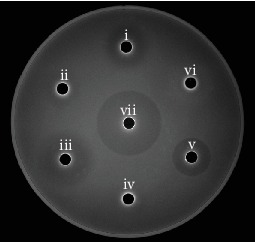
Antibacterial effect to K88 of P25 fermentation broth (FB). i, ii, iii, iv, v, vi, and vii represent the inhibition zones of the FB, FB of acid-free, DL-lactic acid, MRS, Amp, FB of H_2_O_2_-free, and H_2_O_2_, respectively.

The effect of the bacteriostatic experiment is shown in Figure 1. It showed that MRS had no inhibitory effect on K88, but the inhibition of the zone diameter of P25 fermentation broth (FB) was obvious, which indicated that P25 could inhibit the growth of K88. The measured results showed that the inhibition zone of FB (24.58 ± 0.12 mm) accounted for 80% of the positive control ampicillin (30.59 ± 0.45 mm). The inhibition zone of FB after the removal of organic acid was unclear but disappeared when hydrogen peroxide was added. These results suggested that the bacteriostasis of K88 was related to the organic acid and hydrogen peroxide produced by P25.

The pH of FB cultivated for 12 h was about 3.31; it was significantly lower than the initial pH of 7.44 (*P* < 0.05). After 24 hours of cultivation, the pH decreased to 3.29. These results indicated that P25 is a capable producer of acid. The viability count of P25 was still over 10^6^ CFU/mL after incubation in medium with a pH of 2.5 or 0.3% and 0.6% bile salt for 2 h (Table 3). In summary, the in vitro probiotic performance of P25 was exceptional.

### 3.2. *P*. *acidilactici* P25 Prolonged the Lifespan of Worms under ETEC K88 Infection

**Figure 2:**
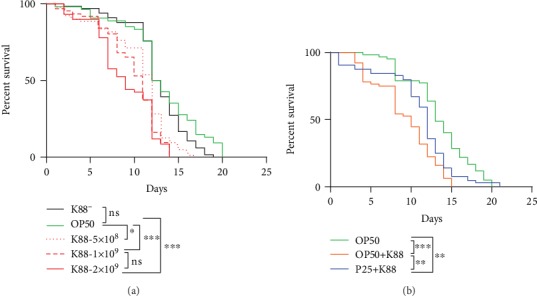
(a) Survival curves of *C*. *elegans* infected with K88 of different concentrations. The number behind K88 represents the concentration of bacteria. (b) Survival curves of P25 against K88-infected *C*. *elegans*. Asterisks indicate significant differences (^∗^*P* < 0.05, ^∗∗^*P* < 0.01, and ^∗∗∗^*P* < 0.001); ns: not significant (same as below).

As shown in Figure 2(a), log-rank (Mantel-Cox) test results showed that the mutant strain K88 (*estB^−^*, *elt^−^*) had no effect on nematode death compared with OP50 (control group, CK) at a concentration of 2 × 10^9^ CFU/mL (*P* > 0.05). However, a significant difference (*P* < 0.05) was observed between nematodes fed 5 × 108 CFU/mL of K88 (attacked group, AT) and CK. These results showed that ST and LT were related to death in nematodes. As the K88 concentration increased, the median survival was gradually shortened. The AT concentration reached at 2 × 109 CFU/mL, after 9 days. Meanwhile, the lifespan of *C*. *elegans* was shortened to 13 days. Thus, a K88 concentration of 2 × 109 CFU/mL was selected for attacking *C*. *elegans*.

The protective effect of P25 to *C*. *elegans* was evaluated (Figure 2(b)). Log-rank (Mantel-Cox) test results showed a significant (*P* < 0.01) difference between the P25 protection group (PR) and AT. On the other hand, the percent survival of nematodes in PR was higher than that in AT from day 4 to day 21. Also, the lifespan and median survival of nematodes in PR were extended by 6 and 2 days, respectively. These results suggested that P25 prolonged the lifespan of *C*. *elegans* under K88 infection.

### 3.3. *P*. *acidilactici* P25 Reduced ETEC K88 Colonization in the Intestinal Tract of Nematodes

**Figure 3:**
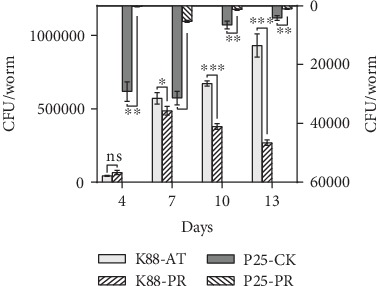
Effect of P25 on colonization of K88 in the intestinal tract of *C*. *elegans*. K88-AT, P25-CK, K88-PR, and P25-PR represent each species bacterial quantity colonized in the intestine of *C*. *elegans* in the attacked group, control group, and protection group, respectively. The results are expressed as the mean ± standard error.

Because the percent survival of nematodes in PR was higher than that in AT after the 4^th^ day of K88 infection, the colonization of bacteria in the intestines of *C*. *elegans* was measured from that point forward. Throughout the experimental period, P25 in nematodes varied from 10^2^ to 10^5^ CFU/mL per nematode. This indicated that P25 successfully colonized the intestinal tracts of nematodes. On the 4^th^ day, the number of K88 in AT and PR was not significantly different (*P* > 0.05). From the 4^th^ to 7^th^ day, the number of K88 increased whether or not the host was fed P25. However, the proliferation of K88 was faster in AT and the abundance of K88 in worms of PR was significantly lower than that of AT (*P* < 0.05). From day 7, the number of K88 in PR began to decline, also meaning that P25 had reached the maximum value of 10^4^ CFU/mL per nematode, but K88 in AT continued to grow. On the 10^th^ and 13^th^ days, K88 in AT were 43.52% and 71.10% higher than those in PR (*P* < 0.01 and *P* < 0.001, respectively). These results indicated that P25 persisted in the intestinal tract of the nematode throughout the experimental period and inhibited K88 colonization (Figure 3).

### 3.4. *P*. *acidilactici* P25 Downregulated the Abundance of ETEC K88 Enterotoxin Genes Both In Vivo and In Vitro

**Figure 4:**
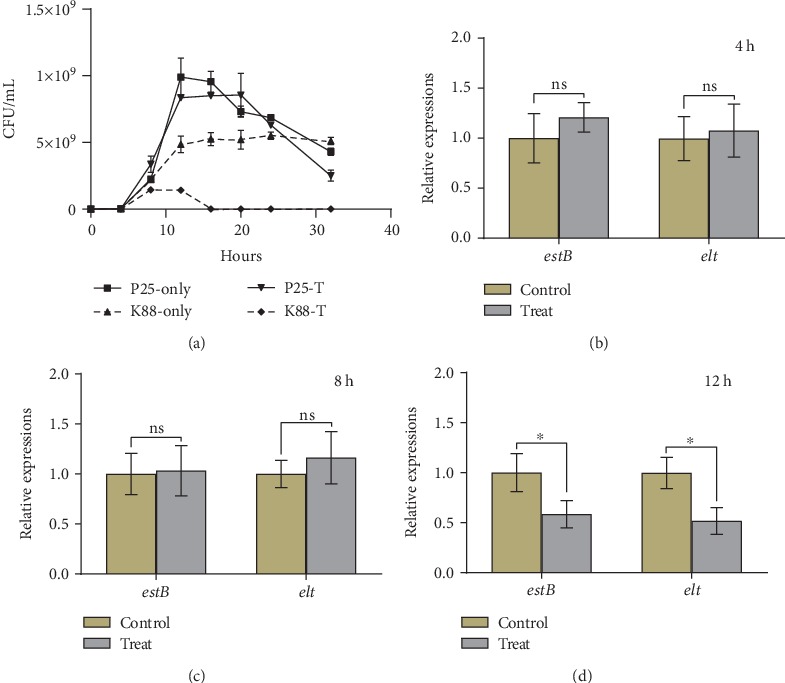
(a) Growth curves of P25 and K88 mixed culture. P25-only, K88-only, P25-T, and K88-T represent P25 and K88 cultured separately and mixedly, respectively. (b–d) Abundance of enterotoxin genes *estB* and *elt* at 4, 8, and 12 h. Control and treat indicate K88 culture alone and mixed culture of K88 and P25, respectively.

In the previous section, we found that P25 significantly prolonged the lifespan of K88-infected nematodes. Considering the pathogenic role of ETEC enterotoxin, the abundance of the K88 enterotoxin genes *estB* and *elt* in vivo and in vitro of *C*. *elegans* was measured to verify whether the protective effect was related to the expression of the enterotoxin gene. K88 and P25 were cocultured, and the abundances of *estB* and *elt* within 12 h were determined. There were no significant differences in the expression of *estB* and *elt* between the mixed-culture group (treat) and the K88 culture alone (control) at 4 h and 8 h (*P* > 0.05) (Figures 4(b)–4(d)). However, at the 12 h point, the abundance of *estB* and *elt* in treat was significantly lower than that in the control (*P* < 0.05), which indicated that the abundance of *estB* and *elt* genes could be inhibited by P25 in vitro. In addition, the growth situation of bacteria was recorded. Under mixed-culture condition, P25 was always more abundant than K88 and the maximum value was 8.35 × 10^8^ CFU/mL at 12 h, but the maximum number of K88 was only 1.42 × 108 CFU/mL, which was significantly lower than the control (4.9 × 108 CFU/mL, *P* < 0.05) (Figure 4(a)). These results showed that P25 not only inhibited the growth of K88 but also inhibited the expression of enterotoxin genes in vitro.

**Figure 5:**
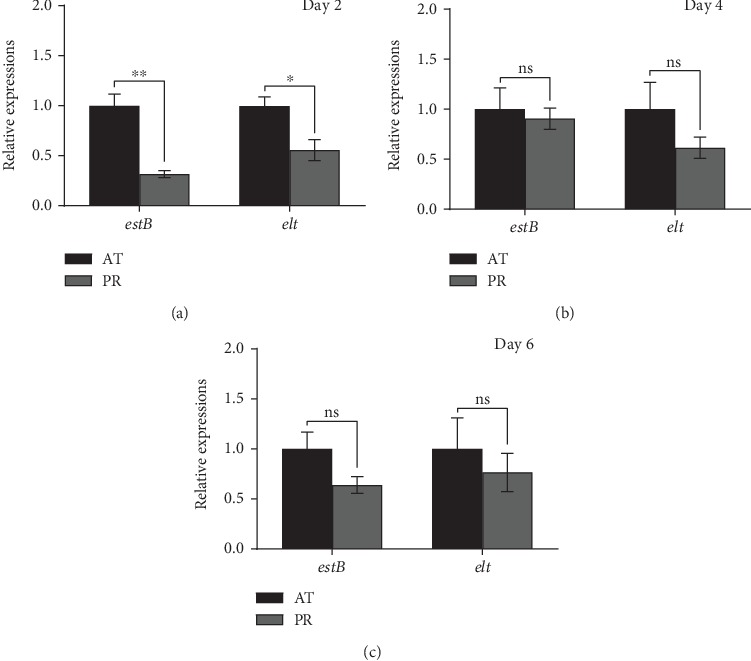
Effect of P25 on the abundance of enterotoxin genes in the intestine of *C*. *elegans*. (a–c) Abundance of K88 enterotoxin genes *estB* and *elt* in the attacked group and P25 protection group on days 2, 4, and 6.

The expression patterns of enterotoxin genes in the intestinal tract of *C*. *elegans* are shown in Figure 5. On the 2^nd^ day of infection, the abundance of *estB* and *elt* in PR was 2.17 and 0.79 times lower than that in AT (*P* < 0.01 and *P* < 0.05), respectively. On days 4 and 6, there were no differences observed in either group (*P* > 0.05), but the abundances of *estB* and *elt* in PR were always lower than those in AT. The above results suggested that the inhibitory effect of P25 on *estB* and *elt* may primarily occur during the initial stages of infection in vivo (Figure 5).

### 3.5. Illumina Sequencing and Read Assembly

Sequencing of worms from each different treatment PR, AT, and CK were performed in triplicate. Approximately 426,214,134 clean reads (150 bp) were obtained after filtering out the linker and low-quality bases in each sample. The data of each sample represented nearly 4 × 10^8^ bp; Q20 and Q30 were greater than 98.5% and 94.7%, respectively, in all samples (Supplementary [Supplementary-material supplementary-material-1]).

### 3.6. Analysis of Gene Expression

**Figure 6:**
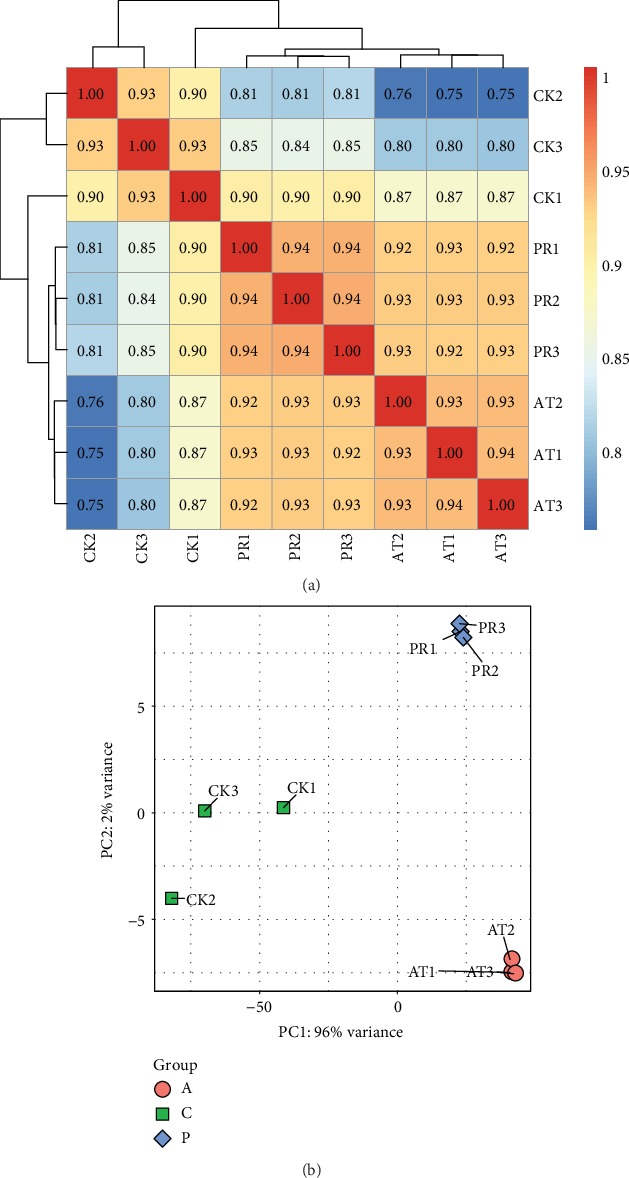
(a) Heat map of hierarchical clustering for gene expression levels in nine samples. The darker the color is, the greater the correlation is. (b) Principal component analysis (PCA) of nine samples. The closer the points of the same color are, the better the aggregation is.

A total of 420,909,912 reads were obtained after removing the reads in the aligned ribosomal database and comparing with the reference genome of *C*. *elegans*. The percentage of reads on both ends of the ribosome reference sequence of all samples was less than 0.01% (Supplementary [Supplementary-material supplementary-material-1]), and the total alignment rate with the reference genome of *C*. *elegans* was greater than 98.6% (Supplementary [Supplementary-material supplementary-material-1]). The saturation and read homogeneity distribution analysis of the sequencing showed that the sequencing amount of the sample reached the saturation standard, and the overall homogenization degree was relatively high (Supplementary [Supplementary-material supplementary-material-1]). Finally, a total of 20,577 genes from 9 samples were obtained, with the longest gene composed of 39,303 bp and the shortest gene of 17 bp. The results of a hierarchical clustering analysis of these genes in the 9 samples found that the correlation indices of the genes in different treatments were different, but the differences within the groups were small (Figure 6(a)); the correlation indices are CK 0.93~1.00, PR 0.81~0.90, and AT 0.75~0.87, respectively. PC1 and PC2 (principal components 1 and 2) in the results of the PCA analysis explained 96% and 2%, respectively, of the distributions of the different groups. The consistency of the principal components within PR or AT was high, indicating that the similarity between the replicate samples was higher than that between samples from different treatments and that the properties of the samples were different (Figure 6(b)).

### 3.7. Analysis of DEGs

**Figure 7:**
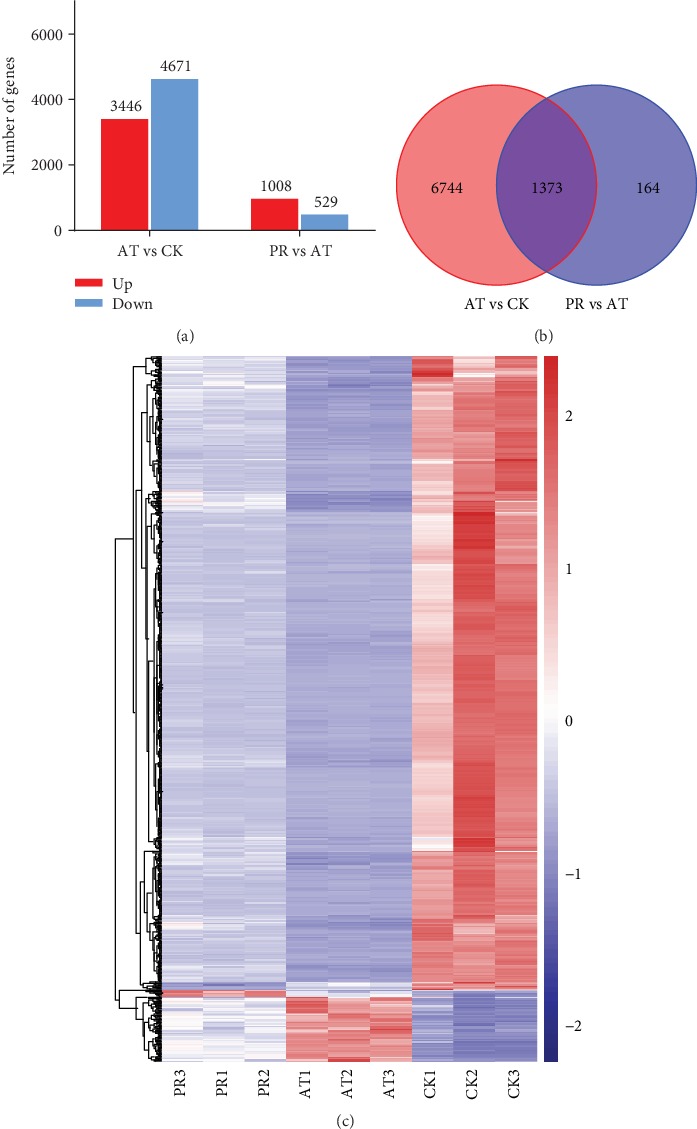
(a) The statistical maps of differentially expressed genes (DEGs) upregulated and downregulated the situation among AT vs. CK and PR vs. AT. (b) Venn diagram comparing the DEGs between AT vs. CK and PR vs. AT. (c) The cluster heat maps of DEGs among CK, AT, and PR. Columns and rows represent samples and genes. Legend represents the log2 (fold change) of gene abundance; the redder the color, the higher the gene expression, and conversely, the bluer the color.

The abundance of genes in each treatment was compared. The results showed that 3,446 upregulated and 4,671 downregulated genes were found in AT relative to CK. Meanwhile, 1,008 upregulated and 529 downregulated genes were found in PR relative to AT (Figure 7(a)). Of which, 1,373 of the differentially expressed genes (DEGs) were common to both of the above DEG pools (Figure 7(b)). Among the identified DEGs, a total of 1,352 showed opposite expression patterns in AT vs. CK and PR vs. AT. Some of these DEGs may be factors induced by P25 that were responsible for extending the lifespan of worms in this study.  Figure 7(c) shows the abundance of all DEGs in each sample. Obviously, the expression patterns of DEGs in PR were closer to CK than AT, which also implied that P25 had a certain protective effect for nematodes against K88.

#### 3.7.1. GO Enrich

**Figure 8:**
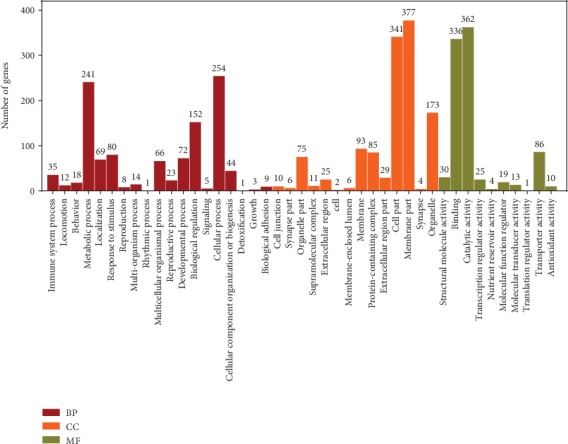
DEGs in GO level 2 of AT vs. CK and PR vs. AT.

**Figure 9:**
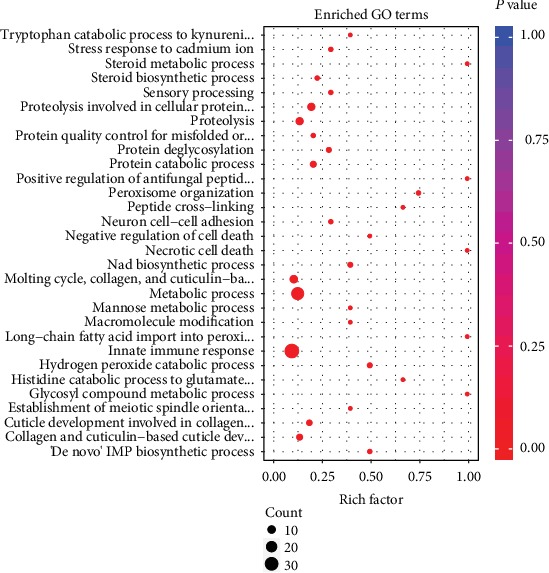
GO enrichment analysis of DEGs in the BP of the AT vs. CK and PR vs. AT (top 30). Count: the bubble size indicates the number of enriched DEGs. *P* value: significant level of enrichment; the redder the color, the higher the enrichment.

When the above 1,373 DEGs were passed through the GO database, they were divided into three principal GO terms of level 2: biological processes (BP, 1,107 genes, 80.63%), cellular components (CC, 1,237, 90.09%), and molecular functions (MF, 886, 64.53%) (Figure 8). Of which, the highest DEGs were involved in the BP of the first 30 GO terms in the AT vs. CK and PR vs. AT groups and were enriched (Figure 9). Of this group, the innate immune responses were the most abundant, followed by metabolic processes and proteolysis involved in the cellular protein catabolic process.  Table 4 lists the top 5 DEGs with the largest differences in expression in the innate immune response GO terms; their encoded products were C-type lectin, UDP-glucuronyl transferase, F-box A protein, multidrug resistance protein pgp-3, and collagen. It can be seen that these DEGs were downregulated when nematodes were infected by K88, and they were upregulated in PR. This suggests that they may play key roles in the responses to K88 in infected nematodes.

#### 3.7.2. DEG KEGG Pathway Analysis

**Figure 10:**
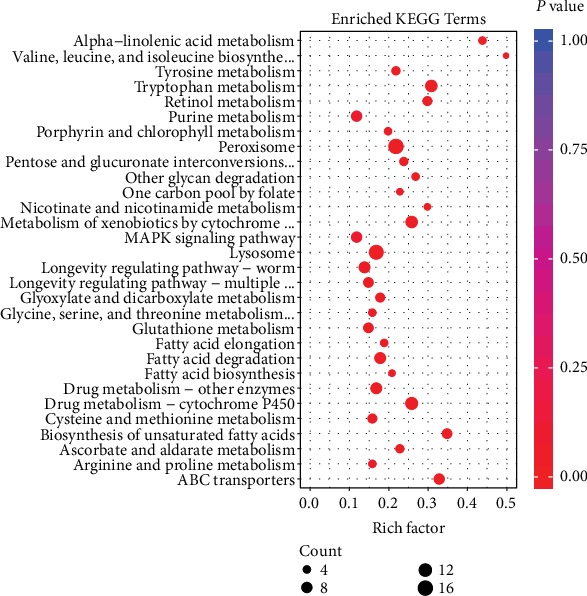
KEGG pathway enrichment analysis of DEGs in PR vs. AT (top 30).

The results of the top 30 pathways identified by enrichment analysis between PR and AT in the KEGG database are shown in Figure 10. Among these pathways, the top three most enriched were peroxisome (with a *P* value of 2.13*E*-05), tryptophan metabolism (*P* value of 3.22*E*-05), and drug metabolism-cytochrome P450 (*P* value of 8.00*E*-05). In addition, the longevity-regulating pathway-worm (LRP-W) (*P* value of 0.032241028) and longevity-regulating pathway-multiple species (LRP-MS) (*P* value of 0.035969185) associated with the lifespan of *C*. *elegans* were enriched. The mitogen-activated protein kinase (MAPK) pathway (*P* value of 0.099829268) associated with immune defense was also found. By comparing the expression of common DEGs in PR vs. AT and AT vs. CK in these 3 pathways, we could see that the genes in each pathway that were downregulated due to K88 infection were all upregulated after incubation with P25, which indicated that these pathways may play a vital role in the defense of *C*. *elegans* against K88 infection (Table 5).

### 3.8. Quantitative RT-PCR (qRT-PCR) to Validate RNA-seq

**Figure 11:**
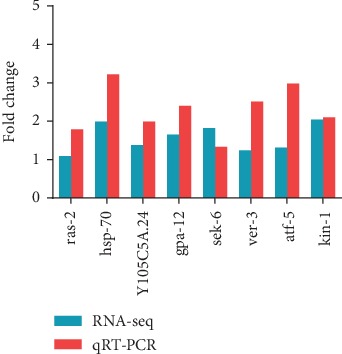
Validation of RNA-seq-based expression patterns using qRT-PCR.

According to the KEGG pathway analysis, the expression patterns of DEGs involved in the MAPK pathway (Table 5) in AT vs. PR were analyzed by qRT-PCR to validate the RNA-seq results. The results indicated that the differential expression patterns of genes relating to the MAPK pathway as measured by RNA-seq analysis followed the same trend in abundances detected by qRT-PCR (Figure 11), which showed that the RNA-seq results were reliable. The differences in fold change in expression can therefore be attributed to differences in experimental treatments and operational errors.

## 4. Discussion

### 4.1. Probiotic Performance of *P*. *acidilactici* P25 In Vitro

A large number of small molecular compounds with bacteriostatic function are produced by LAB during the fermentation process; these molecules include organic acids, hydrogen peroxide, diacetyl, and bacteriocin [52–55]. The present study showed that P25 has a good resistance to K88 due to its ability to secrete organic acid. When P25 was cultured for 24 h, the pH of fermentation broth decreased to about 3.29, and almost all pathogenic bacteria cannot grow at this pH value. P25 also showed a high tolerance under the low pH = 2.5 and high concentration of bile salt (0.3~0.6%) stress treatment. The majority of LAB experienced limited growth under acidic conditions, and CFU values usually decrease by 1~6 orders of magnitude under when subjected to sufficiently low pH conditions [56]. However, the acid resistance of P25 was exceptional; the viability count remained at over 10^6^ CFU/mL during our experiment. In general, the bile salt content in the digestive tract is 0.03%~0.30% [57], but P25 maintained high activity in the high bile salt treatment. These results show that P25 meets the requirements for good probiotic performance.

### 4.2. Effect of *P*. *acidilactici* P25 on the Lifespan of *C*. *elegans*

Due to the high homology of genes between nematodes and mammals and because nematodes have a relatively complete digestive system, *C*. *elegans* can be used as a host-pathogen interaction model in scientific research [58, 59]. In this study, results demonstrated that nematodes treated with P25 had a significant resistance to infection by K88 through multiple mechanisms.

P25 could colonize in the intestinal canal of nematodes. The colonization of pathogenic bacteria can be highly damaging to the host's gut. The colonization ability of bacteria is related to many factors including pili, intestinal microenvironment, and colonization sites on intestinal cells [60–62]. The present study found that the number of K88 in the intestine of nematodes incubated with P25 was decreased from day 7. Thus, P25 could colonize in the intestinal cells of nematodes and inhibit colonization by K88. Qin et al. [63] saw a similar result, in that the ability of a *P*. *acidilactici* to colonize in the jejunum of finishing pigs surpassed that of *E*. *coli*, which indicated that *P*. *acidilactici* does have an antipathogenic bacterial adhesion effect, which supports our results. In addition, there were also some reports that LAB inhibited the colonization by ETEC K88 and *Salmonella enterica* in other cell models, such as *Lactobacillus casei* and *Lactobacillus plantarum* [64, 65]. Zhou et al. [43] reported the colonization of two *Lactobacillus* strains in the intestinal tract of *C*. *elegans*, but they were not affected by the colonization of JG280. In contrast, P25 had a certain anti-ETEC K88 colonization effect. Therefore, our results could also further supplement that the inhibition of pathogens by LAB may be species diverse in host cell colonization.

Another main virulence factor of ETEC is enterotoxins [66]. Previous studies have found that *Lactobacillus casei* inhibited the growth and virulence characteristics of foodborne enteropathogenic enterohaemorrhagic *E*. *coli* (EHEC), *Salmonella typhimurium*, and *Listeria monocytogenes* under mixed-culture conditions [67]. In the present study, when P25 was mixed cultured with K88, not only the growth of K88 was significantly inhibited (*P* < 0.05) but also the abundance of the enterotoxin genes *estB* and *elt* (Figure 4(d)). The expression of these two genes in nematodes was significantly reduced in the initial stage of infection in nematodes with P25. Secondary metabolites such as organic acids, hydrogen peroxide, and bacteriocin are the primary antibacterial agents produced by LAB [53–55]; as mentioned above, the inhibition of K88 by organic acids or hydrogen peroxide generated in P25 fermentation broth has been previously confirmed, and it is possible they are the reason for the decrease of enterotoxin gene abundance.

### 4.3. Transcriptomic Analysis of *C*. *elegans*

#### 4.3.1. Innate Immune Response

*C*. *elegans* has no adaptive immunity or immune cells; therefore, its innate immunity provides the main immune response against pathogen invasion. Our sequencing results suggested that the genes' abundance involved in the innate immune response of *C*. *elegans* fed with P25 or not was completely opposite, compared with the control. Some genes' abundances related to the innate immune response in *C*. *elegans* were significantly reduced due to K88 infection, but this was reversed by P25. Furthermore, the median survival of *C*. *elegans* in PR was significantly extended than AT, also indicating that P25 increased the innate immune response in *C*. *elegans*. These DEGs play an important role in the protection of nematodes against infection. C-type lectins (CTLs) involve in serum glycoprotein homeostasis, pathogen detection, and initiation of immune responses [68]. Clec-39 and Clec-49 protein-deficient nematodes became sensitive to *Serratia marcescens* infection [69]. F-box proteins (FBPs) contribute to cell proliferation, cycle, apoptosis, migration, invasion, metastasis [70, 71], and antiretroviral function [72, 73]. UDP-glucuronyl transferase (UGT) and collagen are mainly involved in chemical detoxification and innate immune responses that have suffered stratum corneum injury [74, 75]. Therefore, P25 may enhance the innate immune response of nematodes to defend against K88 infection and prolong their lifespan.

#### 4.3.2. Longevity-Regulating Pathway

In our sequencing data (Table 5), many genes located at a key node of *C*. *elegans* autophagy (ID: cel04212) were detected. Metallothioneins (MTs, *mtl-1* and *mtl-2*), which are involved in metal detoxification, homeostasis, protection from oxidative stress, and defense against toxic challenges in humans [76], are critical to the longevity of organisms [76]; mice that lack these genes were short-lived [77]. The MnSOD (*sod-3*) systems in *C*. *elegans* fine-tune the insulin-like signaling-based regulation of longevity by acting as physiological redox-signaling modulators [78]. Catalase (CAT, *ctl-1* and *ctl-2*) has the function of regulating host defense to pathogens, apoptosis, aging, inflammation, tumor formation, and mutagenesis. It has been showed that CAT can extend lifespans by 20% in transgenic mice by increasing the enzyme levels through overexpression in various organelles [79, 80]. Glutathione S-transferase (GST, *gst4*) was shown to protect cells from chemically induced toxicity and stress [81]; the knockdown of five GST synthesis-related genes in nematodes can shorten the lifespan of worms [82]. Overall, the increase in abundance of genes related to the longevity-regulated pathway can be attributed to the influence of P25 and extended the life of worms.

#### 4.3.3. MAPK Signaling Pathway

Both transcriptome sequencing and RT-qPCR validation indicated that the expression of some encoding genes of important signaling molecules in the MAPK signaling pathway was upregulated in nematodes incubated with P25. Previous studies have shown that protein kinase A (PKA, *kin-1*) regulates various physiological activities involved in substrates through phosphorylation [83, 84]. Xiao et al. [85] found that PKA resisted *Salmonella* infection by regulating expression of a panel of antimicrobial effectors in nonneuronal tissues [85]. In addition, PKA also regulates the expression of lysosomal genes during intestinal infections. The autolytic signaling molecule produced by the lysosomal pathway, a downstream effector of PKA signaling, participates in the defense against *Salmonella* infection by controlling autophagy. In the present study, the evidence implied that PKA may be a “top priority” when responding to K88 attack. Heat shock protein 70 (HSP70, *hsp-70*) is also an important element in various immune processes, involved in antigen presentation and activation of immune activity and bioadaptation to stress [86, 87]. There are two types of receptor protein tyrosine kinases (PTK, *ver-3*) in the cell: transmembrane receptor PTK and nonreceptor PTK (NPTK), the latter of which has been shown to be a key component of immune system regulation [88, 89]. So, the upregulation of the MAPK signaling pathway by P25 may also be essential to its protective effect against K88 in *C*. *elegans*.

## 5. Conclusions

The results of this study highlight the inhibitory effect of *Pediococcus acidilactici* P25 on enterotoxigenic *Escherichia coli* K88 and explored its inhibitory mechanism. P25 has good probiotic performance, which effectively inhibited the growth in vivo and in vitro and reduced the colonization in the intestinal tract of nematodes of K88. Transcriptome data also revealed that the increased lifespan of K88-infected nematodes may be based on the effect of P25 on their longevity signaling pathways and immune-related pathways.

## Figures and Tables

**Table 1:** The quantitative primers for ETEC K88 enterotoxin gene.

Primer	Sequence (5′⟶3′)
gapA-F	TCCGTGCTGCTCAGAAACG
gapA-R	CACTTTCTTCGCACCAGCG
estB-F	TGCCTATGCATCTACACAAT
estB-R	CTCCAGCAGTACCATCTCTA
Q-elt-F	AGGAGGTTTCTGCGTTAGGTG
Q-elt-R	TTGGTGATCCGGTGGGAAAC

**Table 2:** The primers for transcriptome sequencing result verification.

Primer	Sequence (5′⟶3′)
Actin-F	CCCCACTCAATCCAAAGGCT
Actin-R	GTACGTCCGGAAGCGTAGAG
kin-F1	ACCAGAATACTTGGCACCCG
kin-R1	AGTGCGACGGGAATTTCACT
hsp-F1	CCGGTTGAAAAGGCACTTCG
hsp-R1	TGCACCAAAGGCTACTGCTT
Sek-F2	ACGACGCCCATCTTTATCCG
Sek-R2	TCCAGCAATCACCCTCACTG
Gpa-F1	CTCGGATCCGGTGAATCTGG
Gpa-R1	AGCATCTAACAACACCCGCA
Un-F1	CCAGCAGCCATCAAATACGC
Un-R1	ATCGGCCACACATCCACAAT
atf-F1	GTGCGAAGAAATCGAGCGTC
atf-R1	ACCTGATCCTTCAGCTTGCC
ver-F1	TTACCGTCCGTGAACCTTCG
ver-R1	AAATGTGCCACCTACGCTGA
ras-F1	GTTTCGGAGCAAGAGGGACG
ras-R1	TTGGTTCGTGGGACAGATGC

**Table 3:** Antibacterial, acid production, acid and bile salt tolerance performance of P25.

Bacteriostasis (mm)	FB	Acid-free	DL-LA	MRS	Amp	H_2_O_2_-free	H_2_O_2_
	24.58 ± 0.12^d^	15.33 ± 0.46^e^	35.38 ± 0.14^b^	<0.01^f^	30.59 ± 0.45^c^	<0.01^f^	38.85 ± 0.09^a^
pH of FB		0 h		12 h		24 h	
		7.44 ± 0.07^a^		3.31 ± 0.03^b^		3.29 ± 0.02^b^	

Antiacid (log(CFU/mL))		0 h				2 h	
		6.97 ± 0.01				6.35 ± 0.01	

Antibile salt (log(CFU/mL))		Control		0.3% bile salt		0.6% bile salt	
		6.46 ± 0.01		6.28 ± 0.02		6.13 ± 0.01	

The letters in the upper right corner of the number indicate significant difference (*P* < 0.05) (same as below). <0.01 is showed when the diameter of the inhibition zone was less than the well.

**Table 4:** Common DEGs of innate immune response in AT vs. CK and PR vs. AT (top 5).

Gene_ID	Annotation	Log2FC	*P* value	FDR
NM_068048.4	C-type LECtin	-7.14/2.47	2.33*E*-44/5.73*E*-12	2.76*E*-42/1.83*E*-09
NM_069532.4	UDP-GlucuronosylTransferase	-4.33/1.86	3.30*E*-43/2.52*E*-10	3.23*E*-41/5.07*E*-08
NM_064966.1	F-box A protein	-5.57/1.43	1.21*E*-42/6.13*E*-07	1.08*E*-40/3.16*E*-05
NM_077500.4	Multidrug resistance protein	-4.28/1.14	1.99*E*-40/1.44*E*-06	1.36*E*-38/6.21*E*-05
NM_077469.6	COLlagen	-4.28/1.13	5.51*E*-39/4.86*E*-06	3.17*E*-37/0.000164

Gene ID: GO function ID. Log2FC: a value greater than 0 represents an upregulation of gene expression and less than 0 indicates a downregulation. FDR: correction of the *P* value multiple hypothesis test.

**Table 5:** DEGs of LRP-W, LRP-MS, and MAPK pathways in PR vs. AT and AT vs. CK.

Pathway	Count	Down	Up	Name of genes
LRP-W	8	0/8	8/0	*mtl-1*, *daf-36*, *fat-5*, *atf-5*, *gst-4*, *sod-3*, *ctl-1*
LRP-MS	6	0/6	6/0	*kin-1*, *hsp-70*, *aakb-1*, *sod-3*, *ctl-1*,*2*
MAPK	8	0/8	8/0	*kin-1*, *hsp-70*, *sek-6*, *gpa-12*, *Y105C5A*.*24*, *atf-5*, *ver-3*, *ras-2*

Down and up represent the gene expression trend in PR vs. AT and AT vs. CK.

## Data Availability

The data used to support the findings of this study are available from the corresponding author upon request.

## Supplementary Materials

Supplementary Materials Tables S1~S3: clean reads, the percentage of reads on both ends of the ribosome reference sequence, and the total alignment rate with the reference genome of transcriptome sequencing of nematodes. Figures S1 and S2: the saturation and read homogeneity distribution analysis of the sequencing. Transcriptome data can be found at https://dataview.ncbi.nlm.nih.gov/object/PRJNA588132.
