# Supplementary material for: Pediococcus acidilactici P25 Protected Caenorhabditis elegans against Enterotoxigenic Escherichia coli K88 Infection and Transcriptomic Analysis of Its Potential Mechanisms
Source: Biomed Res Int. 2020 Mar 30;2020:7340312. doi: 10.1155/2020/7340312 (PMC7150717; doi:10.1155/2020/7340312)
Supplement: Supplementary Materials — Tables S1~S3: clean reads, the percentage of reads on both ends of the ribosome reference sequence, and the total alignment rate with the reference genome of transcriptome sequencing of nematodes. Figures S1 and S2: the saturation and read homogeneity distribution analysis of the sequencing. Transcriptome data can be found at https://dataview.ncbi.nlm.nih.gov/object/PRJNA588132. [file 7340312.f1.docx]

Table S1: Statistics of transcriptome data filtering

| Sample | Clean pair Reads | Clean Base(G) | Q20(%) | Q30 (%) | GC Content (%) |
| --- | --- | --- | --- | --- | --- |
| CK1 | 23157015 | 6.94 | 98.74 | 95.38 | 46.57 |
| CK2 | 24122236 | 7.22 | 98.53 | 94.76 | 46.24 |
| CK3 | 22063080 | 6.61 | 98.63 | 95.07 | 46.40 |
| AT1 | 19628897 | 5.88 | 98.72 | 95.35 | 47.51 |
| AT2 | 19071273 | 5.71 | 98.71 | 95.43 | 47.41 |
| AT3 | 19926813 | 5.96 | 98.64 | 95.18 | 47.49 |
| PR1 | 22021735 | 6.6 | 98.78 | 95.51 | 47.28 |
| PR2 | 28206864 | 8.45 | 98.72 | 95.33 | 46.74 |
| PR3 | 34909154 | 10.46 | 98.73 | 95.38 | 47.06 |

**Clean pair Reads:** reads of filtering out the linker and low-quality bases. **Clean Base:** the total number of bases filtered, ie the number of clean reads × length. **Q20:** bases with correct recognition rates above 99%. **Q30:** bases with correct recognition rates above 99.9%. **GC Content:** number of G+Cs as a total number of bases percentage.

Table S2: Statistics of rRNA alignment

| Sample | Total paired reads | Paired mapped reads | Unpaired mapped reads | Unmapped reads | Total mapped |
| --- | --- | --- | --- | --- | --- |
| CK1 | 23157015 | 1756(0.01%) | 72(0.00%) | 23155188(99.99%) | 1828(0.01%) |
| CK2 | 24122236 | 2710(0.01%) | 92(0.00%) | 24119434(99.99%) | 2802(0.01%) |
| CK3 | 22063080 | 2106(0.01%) | 91(0.00%) | 22060883(99.99%) | 2197(0.01%) |
| AT1 | 19628897 | 1493(0.01%) | 92(0.00%) | 19627312(99.99%) | 1584(0.01%) |
| AT2 | 19071273 | 949(0.00%) | 200(0.00%) | 19070124(99.99%) | 1149(0.01%) |
| AT3 | 19926813 | 950(0.00%) | 42(0.00%) | 19925822(100.00%) | 992(0.00%) |
| PR1 | 22021735 | 865(0.00%) | 56(0.00%) | 22020814(100.00%) | 921(0.00%) |
| PR2 | 28206864 | 883(0.00%) | 48(0.00%) | 28205932(100.00%) | 932(0.00%) |
| PR3 | 34909154 | 1180(0.00%) | 75(0.00%) | 34907899(100.00%) | 1255(0.00%) |

**Total paired reads:** number of Clean paired reads. **Paired mapped reads:** percentage of Reads on both ends of the reference sequence. **Unpaired mapped reads:** Reads at one end of the reference sequence. **Unmapped reads:** Reads do not match the reference sequence. **Total mapped:** comparison rate.

Table S3: Statistics of comparing with reference genome

| Sample | Total paired reads | Paired mapped reads | Unpaired mapped reads | Unmapped reads | Total mapped |
| --- | --- | --- | --- | --- | --- |
| CK1 | 23134369 | 22656379(97.93%) | 166010(0.72%) | 311980(1.35%) | 22822388(98.65%) |
| CK2 | 24119365 | 23653334(98.07%) | 201633(0.84%) | 264398(1.10%) | 23854967(98.90%) |
| CK3 | 22054321 | 21621006(98.04%) | 171363(0.78%) | 261952(1.19%) | 21792369(98.81%) |
| AT1 | 19627238 | 19211113(97.88%) | 142071(0.72%) | 274054(1.40%) | 19353184(98.60%) |
| AT2 | 19046402 | 18636028(97.85%) | 150256(0.79%) | 260118(1.37%) | 18786284(98.63%) |
| AT3 | 19910237 | 19513004(98.00%) | 153040(0.77%) | 244192(1.23%) | 19666044(98.77%) |
| PR1 | 22020774 | 21623644(98.20%) | 159765(0.73%) | 237365(1.08%) | 21783409(98.92%) |
| PR2 | 28205895 | 27673928(98.11%) | 212201(0.75%) | 319766(1.13%) | 27886129(98.87%) |
| PR3 | 34907837 | 34247637(98.11%) | 262544(0.75%) | 397656(1.14%) | 34510182(98.86%) |


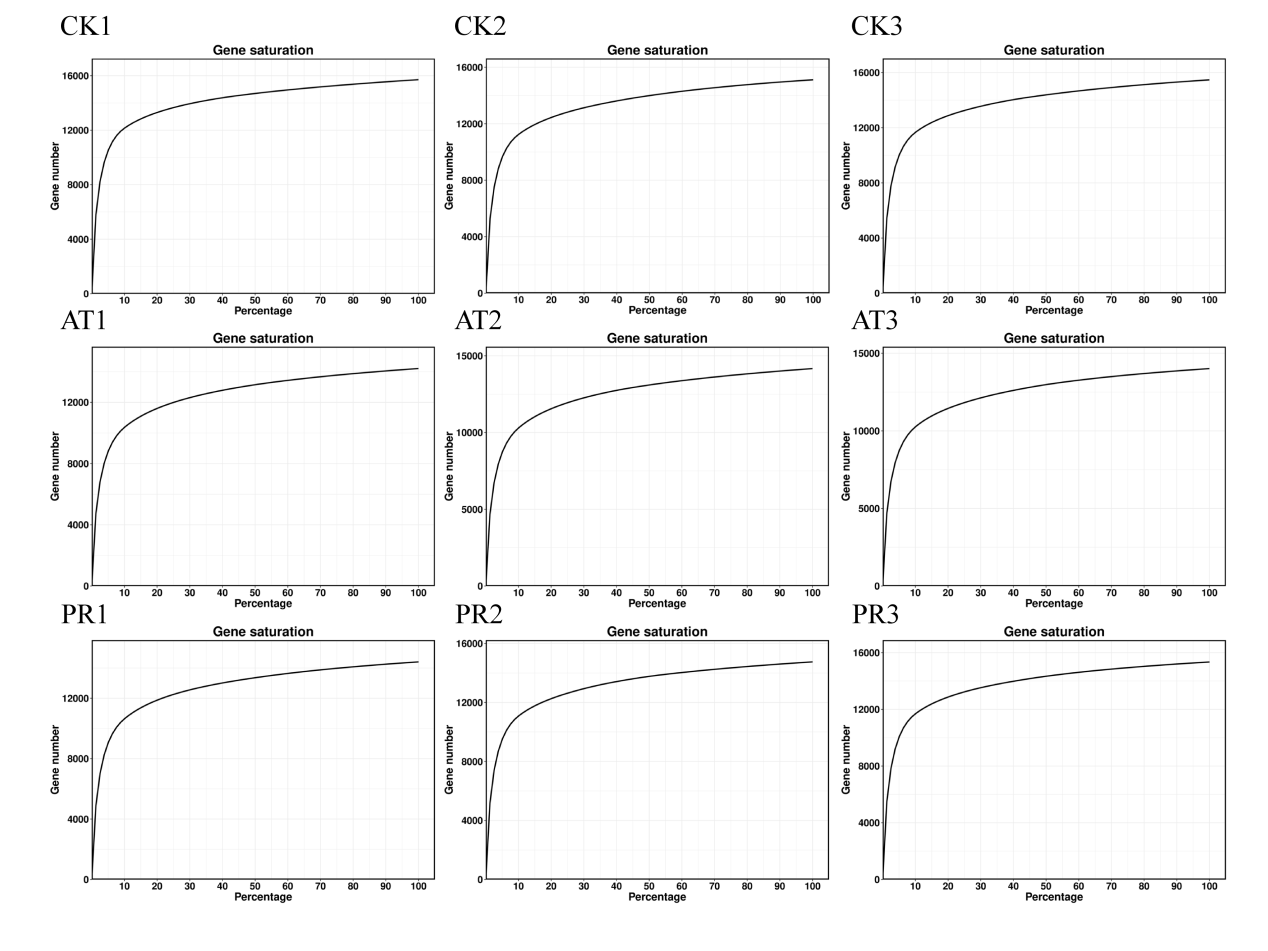


Figure S1: Sequencing saturation curve distribution map.


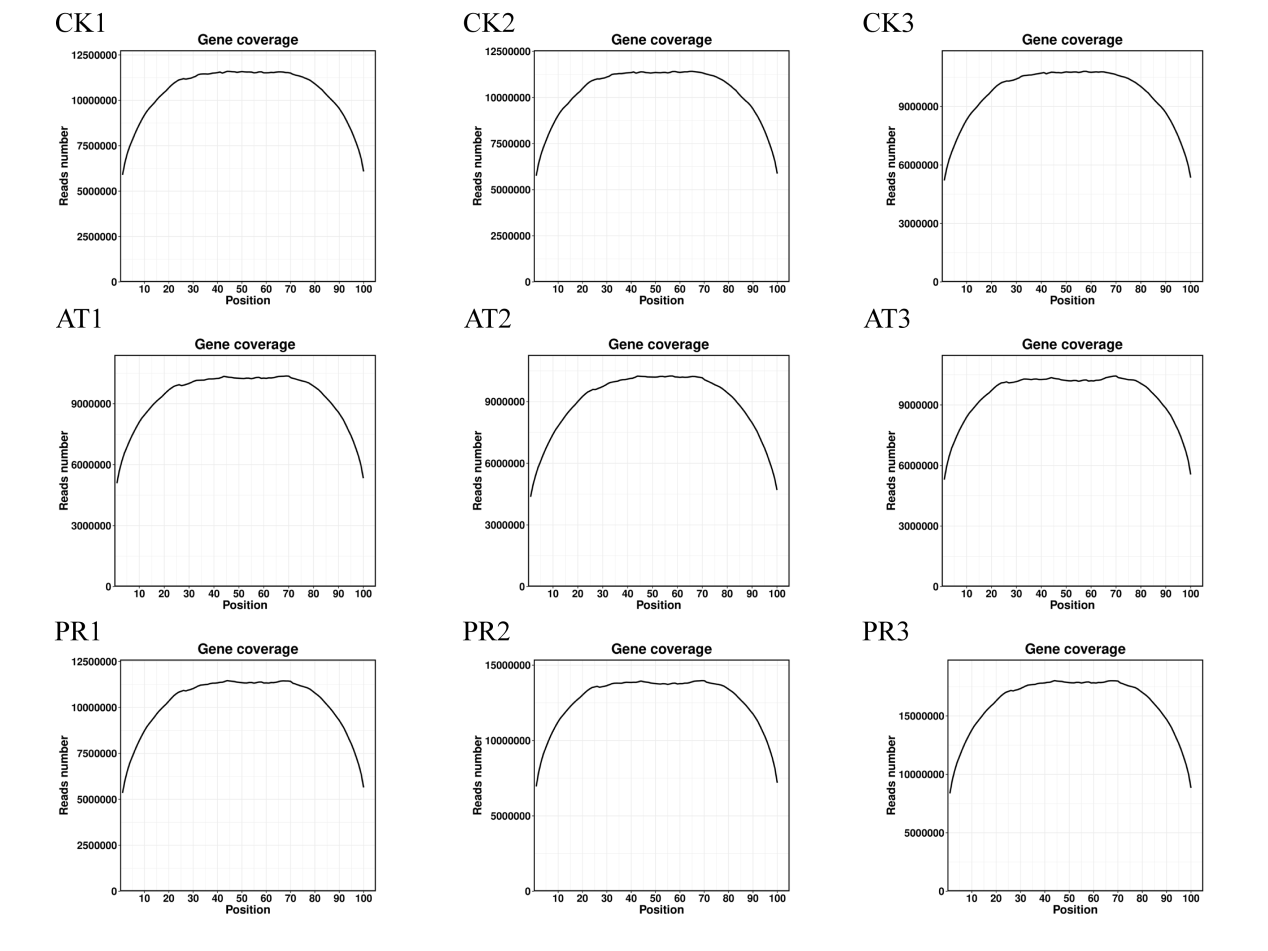


Figure S2: Read distribution uniformity statistics on reference genes.
